# Shifts in FGM/C practice in Sudan: communities’ perspectives and drivers

**DOI:** 10.1186/s12905-019-0863-6

**Published:** 2019-12-30

**Authors:** Nafisa Bedri, Huda Sherfi, Ghada Rudwan, Sara Elhadi, Caroline Kabiru, Wafaa Amin

**Affiliations:** 1 Gender and Reproductive Health and Rights Resource and Advocacy Center, Ahfad University for Women, Khartoum, Sudan; 2 Population Council, Nairobi, Kenya

**Keywords:** Female genital mutilation/cutting, Health care providers, Medicalization, Sudan

## Abstract

**Background:**

Although Sudan has one of the highest prevalence of female genital mutilation or cutting (FGM/C), there have been shifts in e practice. These shifts include a reduction in the prevalence among younger age cohorts, changes in the types of FGM/C, an increase in medicalization, and changes in age of the practice. The drivers of these shifts are not well understood.

**Method:**

Qualitative data drawn from a larger study in Khartoum and Gedaref States, Family and Midwife individual interviews and focus group discussions. Analysis and categorization within a Social Norms theoretical framework.

**Results:**

Major findings confirmed shifts in the type FGM/C (presumably from infibulation to non-infibulating types) and increasing medicalization in the studied communities. These shifts were reported to be driven by social, professional and religious norms.

**Conclusion:**

Changes in FGM practice in Sudan include drivers which will not facilitate abandonment of the practice instead lead to normalization of FGM/C. Yet professionalisation of Midwives including their oath to stop FGM/C has potential to facilitate abandonment rapidly if developed with other Sudan health professionals.

## Background

Female genital mutilation/cutting (FGM/C) is defined as all procedures that involve partial or total removal or injury of the female external genitalia for non-medical reasons [1].

In sub-Saharan Africa and the Arab States around 200 million girls and women were exposed to this harmful practice since 2016 [2, 3].

Female Genital Mutilation/Cutting is a deeply rooted practice held in place by beliefs around controlling women’s sexuality and preserving girls’ sexual purity. It is often started and carried out by grandmothers in the family or social network, fearing social stigmatization and reduced opportunities for marriageability [4]. FGM/C is also practiced as a religious obligation, and associated with cultural beliefs of its role in enhancing the girl’s cleanliness and beauty after removal of unfeminine body parts [5, 6].

Campaigns against FGM/C in Sudan started some fifty years ago, yet, recent statistics show high prevalence of 87% among women at reproductive age 15–49 years and 31% among young girls (< 15 years) [7]. Although the rate of FGM in the population is still high, there have been many changes in the practice. Firstly is the shift in the type of FGM/C practiced, from the (Sudan widely practiced) Type III FGM/C (commonly referred to as the ‘pharaonic cut’) [8] to Type 1 (often called the Sunna cut (a word used for actions underpinning Islamic religious teachings) [9]. It is believed that the focus of anti-FGM/C campaigns on the health risks of FGM/C may have played a key role in driving this change [10]. Moreover, Sunna cuts, being of lesser severity, are believed to avert the health risks associated with more severe forms of the practice.

Secondly, girls in Sudan are having FGM/C at an older age. Data from the most recent Multiple Indicator Cluster Survey (MICS) in Sudan showed an increase in the proportion of women aged 15–49 years, who reported that they were cut at age ten years and older, more than doubling from 10% in 1980–1989 to 23% in 2000–2014 [11]. Further, there was a significant decrease (by nearly two thirds) in the proportion of women reporting that they were cut when they were four years old or less between the years 1989 and 2014 (13 and 5% respectively).

The third change is a shift to health professionals performing FGM/C (medicalization). According to the 2014 Multiple Indicator Cluster Survey (MICS) more than half (58%) of girls (10–14) years in Sudan were circumcised by a health professional [7]. The MICS secondary analysis report showed an increase in medicalization where trained midwife performed 72% of FGM/C between 1990 and 1999 in comparison to a higher 80%in 2000–2014 women aged 15–49 years [11].

The drivers of these changes re not fully understood. Interrogating the shifts in the practice of FGM/C, their drivers, and how they are inter-linked is essential for informing and strengthening FGM/C abandonment interventions. This study therefore sought (across two States od Sudan in a wide range of social groups and across a range of health professionals) toinvestigate on the one hand the drivers of change in the type of FGM/C, on the other hand drivers of medicalization. In addition, it seeks to identify drivers leading to an older age for FGM/C.

### Theoretical framework

There are many theoretical frames which can explain the complicated relationships between the different motives behind the practice of FGM/C.

FGM/C is not only a social convention, it is also a social norm—a rule of behavior that members of a community are expected to follow, and are motivated to follow, through a set of rewards, and negative sanctions [12]. Adhering to social norms helps maintain an individual’s (or family’s) acceptance and social status in the community.

Traditionally in Sudan, all health professionals are highly regarded particularly midwives, in society making them a high influential. To understand the drivers of the shifts in FGM/C among our participants (family members and health professionals). We applied Social Norms theory by examining on the one hand personal attitudes, perceptions and social expectations towards FGM/C (who and what others in reference group do, prefer to do and expect them to do); and on the other hand their views about why people would want medicalized FGM/C; its acceptability (normative experience), and trends they observe in the Type of cutting.

## Methods

### Study design

Data are drawn from qualitative interviews (in-depth interviews and focus group discussions) conducted as part of a larger community-based, cross sectional, comparative mixed methods study [13]. The larger study examined quantitative and qualitative aspects of the shift in the Type of FGM/C, and supply and demand aspects of medicalization (the full study report can be found at http://www.saleema.net/arabic/upload/Medicalisation%20in%20Sudan_final.pdf.

### Study sites

Both Khartoum and Gadaref States are home to a diversity of ethnic groups and have a significant presence of programs aimed at promoting FGM/C abandonment. The two states have midwifery schools, medical schools and each has an Academy of Health Sciences. In addition, midwives in both states are expected to take an oath not to practice FGM/C.

Gedaref, has a state law prohibiting FGM/C, but Khartoum State has no law against the practice.

Medicalisation is widespread in Khartoum State but far less so in Gedaref. In the 2014 MICS, 94% of women aged 15–49 years in Khartoum reported that they had been cut by a health professional (trained midwife or other health professional) compared to 43% among their counterparts in Gedaref [11].

### Sample size and sampling

Families selected for this study had to have at least two daughters who had been cut, with at least five years of age difference and the youngest being younger than ten years of age. Within each family, the mother, father, an older sibling, the girl herself (if older than 18), and grandmother (if available) were interviewed to gain a comprehensive understanding of the perceptions and experiences of different family members. Families were identified from a community mapping survey conducted as part of the larger study and through snowball sampling.

Trained Midwives were recruited for individual in depth interviews. Traditional birth attendants (who had no training and still active providers) were also interviewed in rural Gedaref. Twenty two individual interviews were conducted with the trained midwives and two with the traditional birth attendants.

Family in-depth individual interviews numbered 33 mothers (15 in Khartoum State, 18 in Gedaref State). Thirteen family member focus group discussions were held (5 in Khartoum State and 8 in Gedaref State) with one or two FGD in each State for mothers, fathers, grandmothers and girls over 18. For detailed socio-demographic characteristics of the study participants, please see Additional file 1: Table S1.

### Procedures

In-depth interviews and focus group discussions were held with selected families were collected during the first quarter of 2017 (January–March). Participants were consulted on the selection of the venues for the focus group discussions to provide them with a comfortable and safe environment for them to talk freely. Accordingly, all interviews were conducted in the houses of the respondents. Focus group discussions with men were conducted in different places such mosques, open public playing grounds, and house of a community leader, and with women were conducted in a primary school class and house of a community leader. Participants sat in separate groups to facilitate discussion, and two researchers facilitated the discussion. Discussions and interviews were conducted in Arabic and guided by semi-structured interviews guides. The guide used in interviews contains 12 questions with some probes for each one to assist the researcher. It was used with four family members namely mothers, grandmothers, fathers and girls. The main focus of it was to collect data on types known by the respondents, description of the type(s) mentioned, shift in type, HCP and age, the medical cadre who is practicing FGM/C, the possibility and enhancing factors to abandoning FGM/C in Sudan. (More details in the attached copy of the guide). Interviews and discussions took about 1–2 h and were recorded by tapes. Translation of all transcripts was done after the cleaning process of the transcripts and it was revised by one of the English language lecturers in the School of Languages at Ahfad University for Women. Coding and memo writing were used to analyze the data to create categories that represent the participants’ perspectives. The analysis of the qualitative data started immediately after the completion of the data collection. It started by transcription of audio data, familiarization with the data, developing of themes and codes and finally relating the codes. The qualitative data was double transcribed taking into accounts nonverbal cues. Then the transcripts were read first thoroughly to identify any differences between the two sets, and then categories were identified using content analysis through coding of the data. Some of the codes used: main drivers and factors making families decide to shift in the type of FGM/C, and performer of FGM/C; attitudes of families and health providers towards performing less severe cutting or not cutting at all; and impact of parental education, socio-economic, religion and social pressure on the choices of type, and performer of FGM/C practice.

## Results

### Shift from type III to type, FGM/C and perceived drivers

Reduction in Type III FGM/C is being driven forward by religious teaching, awareness raising sessions in mosques and hospitals, the increasing recognition of complications and recovery time so a safer procedure is needed, and the Oath taken by midwives to stop FGM/C which helps them justify stopping the practice and is also helping communities to stop the practice of Type III. Women pressurized into FGM/C will choose Type 1 if they can. Reduction in Type III FGM/C in Sudan is still being held back by strong cultural commitment to the practice of FGM/C, social networks and reference groups forcing continuation of Type III, and the practice of community social ostracism of trained midwives who refuse to do FGM/C.

Both men and women described Type I as Sunna-a practice approved by religion. Many people, believe erroneously type I is religiously required and approved. Explaining the religious basis for Type I FGM/C, a 30–35 years old respondent who had completed secondary school explained:

*“We do Sunna, which is the Sunna (teaching) of Prophet Mohamed (PBUH) he told UmiAtia [a woman in Prophet Mohamed’s time] not to cut much and leave parts as they are. This is the Sunna. We should do it.”*

Focus group discussion, Fathers, Umbadda

Educational and awareness-raising interventions, especially in urban areas are having an influence on the shift in the Type of FGM/C. Awadia, a 42-year-old, secondary school educated respondent living in Khartoum, explained: *“People used to circumcise (the) Pharaonic (way) (Type III), but nowadays [we have] shifted to Sunna because of the education and awareness sessions in the mosques and hospitals”.*

The perception that Type I has no complications and is *safer* was also reported to drive the shift from Type III. Explaining why people in her community prefer the Type I cut, a 40–45 years old, secondary school educated respondent living in Khartoum State explained that: *“When a girl is circumcised (in the) ‘Pharaonic’ (way) (Type III), she may suffer from bleeding, obstructed labor, and difficult sexual intercourse with the husband.”* Other health-related complications mentioned associated with Type III FGM/C included severe infections, and abscess.

Women and girls who had undergone Type III FGM/C were also noted to need a lengthy recovery period. For some men, such as one father from rural Khartoum State, this lengthy period was disadvantageous to them because it meant that they had to take over tasks normally performed by women. As he explained:

*“The ‘pharaonic’ (Type III FGM/C) causes women great problems. They (have to) stay in bed for 40 days. I have suffered in this situation as I had to do the household chores and also the cooking. I told her (the woman who did the FGM/C) (she should) do Type I only. Women with Type I FGM stay in bed for a week and then move around. The future for women is in Type I.”*

Women who are against FGM/C themselves, but who were pressurised to undergo it by older women relatives reported they would want to have Type 1 as it is less severe.

Importantly the midwifery oath to abandon FGM, now taken by all 3 year trained is seen as the basis for many midwives to say they are now against Type III FGM/C. These midwives may have created an understanding in communities that Type III is illegal. Women in both rural and urban areas noted that many midwives now refused to perform FGM/C, particularly type III, based on the oath.

Reduction in Type III FGM/C in Sudan is still being held back by strong cultural commitment to the practice of FGM/C and social networks and reference groups forcing continuation of Type III.While most mothers noted that they had shifted to practicing less severe FGM/C due to health and religious reasons, a very small number of mothers were insistent about performing Type III FGM/C. These mothers noted that Type III FGM/C has long been a strong cultural practice. As noted by a 25–30 years old respondent who was living in Khartoum noted:

*“Pharaonic circumcision (Type III FGM/C) is very deep in our culture and we will not give it up. We have inherited it from our grandmothers. We will ensure it is done if a mother died, then an aunt or the grandmother will get it done”.*

Social Networks and reference groups are perpetuating severe Type III FGM/C. Mothers often stated that they are forced to do the type of FGM/C that is acceptable to others in their community even when their personal preference is different. As noted by a 35-40 years old, a secondary school educated respondent living in Khartoum State:

*“I really do feel guilty; my four daughters were circumcised (in the Pharaonic (way) (Type III) by my in-laws. I wanted them to have (the) Sunna (Type 1), but my in-laws refused.”*

Increased use of traditional providers of FGM/C who are more likely to continue Type III also occurs because trained midwives refusal to participate in FGM/C means they will become socially excluded. For these midwives, when they refuse to perform FGM/C it can result in ostracism by their community and peers. Negative sanctions against midwives who refused to perform FGM/C were reported by both midwives themselves and community members. As one woman explained:

*“We have to bring the traditional midwife from far away when our local one refuses to do FGM. We bring the dayat aljabal [traditional midwife] to conduct the FGM (‘to cut our daughters’) and when we want help to deliver their babies we bring a relative from Omdurman. The midwife we have here does not cut the girls (she refuses to do the FGM/C) and so we do not talk to her or invite her to anything. She has lived here with us for twenty years but she does not want to do things for us.”* 35-40 years old, primary school educated respondent, rural Khartoum

Despite clear drivers to reduce Type III FGM/C there is a problematic issue with focus on reduction in Type III FGM/C, since some midwives and families reported they believe that Type I is not FGM/C which justifies the practice of FGM/C Type 1 continuing. Even when people claimed to have stopped practicing FGM/C, they were likely to practice Type I.

### Increased medicalization of FGM/C and perceived drivers

Our results in both States confirmed a shift from using traditional midwives to perform FGM/C to using health care providers including trained midwives.

Increase in the Medicalization of FGM/C is being driven by families’ belief it may be safer and more hygienic; the health campaigns that promote the dangers of FGM/C; and strong views that surely a doctor conducting FGM/C would not want to cause harm. In addition, families pressurised socially into having FGM/C may prefer a health care provider to do it. Further drivers of medicalization are health care providers justifying their involvement in FGM/C as response to demand; the widespread availability of health care professionals prepared to do FGM/C; some 3 year trained Midwives and 1 year trained Village midwives thinking Type 1 FGM/C is not FGM/C so it is not against the oath to do it;. Nonetheless there are drivers to reduce medicalization too, with 3-year Midwives who have taken the professional oath to cease FGM/C sticking to their word.

Families reported they choose health care providers because they perform FGM/C under safer and more hygienic conditions.

Health care providers also noted the growing awareness of health hazards of FGM/C. As a midwife from Khartoum State described;

*“In the past people performed this [FGM/C] and they did not know its danger. They used to bring a grandmother who might use a non-sterile razor to do the FGM. She did not know that bleeding might occur, and the girl could have problems. But when people learned (about these risks and complications) they started to go the health centers because they might save their daughter’s life if something went wrong”*

Health campaigns (emphasizing risks of FGM/C) were identified as playing a part in increasing medicalization as people believe that the risks with FGM/C are lower if health care providers perform it.

Mothers said they preferred having doctors perform FGM/C, since they believe doctors are more knowledgeable and will not cause harm to their daughters. A 30–35 years old respondent in the Kriab (urban) area of Khartoum state said;

*“Doctors are knowledgeable and (they) do the right thing. If a doctor says to us, “Do circumcise,” then we will do it because if circumcision (FGM/C) causes harm they surely would not perform it.”*

Perception that doctors are ‘safe performers’ was reported to be driving a new “social norm” that ‘a doctor will do it’.

Some participants’ narratives, suggested that medicalization would increase even more if more female doctors performed FGM/C. A 30–35 years old respondent in rural Khartoum who had completed primary schooling and had two daughters with FGM/C explained;

*“We, Arabs, do not take our girls to male doctors but to the midwives. (We take ) the boys to the male doctors”* Similarly, a 25-30 years old, secondary school educated respondent in Umbadda who had two daughters (one with FGM/C one intact), noted *“We will only let our daughters be seen by a female doctor.”*

Some health care providers explained their practice of FGM/C by being merely responsive to demands for FGM/C by the families, believing that if they did not meet these families’ demands for FGM/C, they will resort to traditional practitioners and end up with more severe FGM/C Type III.

Other factors for the increase in medicalization include changes in people’s reference groups and social networks due to migration, as well as the availability of practicing health care providers. Some participants also noted that women who were against FGM/C, but who were forced to undergo the procedure, would resort to medicalization.

As such, there was a general belief that if health care providers abandon FGM/C, many people would abandon the practice, resulting in less demand.

### Shift in age of FGM/C and perceived drivers

Older age change drivers found included increasing awareness of raised health risks of FGM/C to a younger girl. Yet an opposing belief was that early age FGM/C could cure illness.

Fewer participants referred to a change in the age of cutting. The direction of the shift was not consistent. In Gedaref State, it was noted that there was an increase in age of performing FGM/C from five years to 11 or 12 years, probably driven by the belief it will lead to quicker healing, and show of maturity so that they could get husbands. Whereas, some mothers mentioned they prefer younger age of cutting, such as three years, as a ‘treatment’ of illness. Illustrating this view, a 26-year-old respondent explained:

*“We do not normally circumcise (do FGM/C) at a young age, but I did my elder daughter at the age of 3 years because she was sick. The people told me FGM/C would make her well … … and she got better.*

The age of FGM/C varies by ethnicity in Sudan. Among the Meloha in Gedaref State, daughters have FGM/C immediately after birth, or a week later during the “*Semaia”* celebration party or *“Seboo*” (the naming ceremony). Often, the FGM/C cutter is the same provider who delivered the baby. In Gamoeia, in Khartoum State, on the other hand, FGM/C is performed at the age of six to ten years reportedly because girls this age heal faster and face fewer risks. A 25–30 year-old, primary school educated respondent in urban Gederaf stated in the focus group discussion: “*I prefer to circumcise my daughters at an older age to prevent her having more pain and more problems”.*

## Discussion

The results of our study indicated a clear shift less severe Type I of FGM/C and is continued by the involvement of health care providers and the deep religious conception attached to FGM/C Type 1, being more often the prevalent reason for the increased prevalence of Type I in Sudan by literature [14]. However, in our study, a number of new drivers are importantly identified. The issue of awareness raising by health care providers individually and in hospitals as well as awareness raising sessions in mosques, and the Oath taken by Midwives, all encouraging families to prefer the less severe Type of FGM/C believing it is less harmful pose novel exposes new scopes on the practice. These shifts may also stem from the ongoing and continued national campaigns that highlight the health risks of FGM/C, and the consequences and the severity of Type III. The new findings of drivers that are maintaining practice of FGM/C Type III are important. Reduction in Type III FGM/C in Sudan is still being held back by strong cultural commitment to the practice of FGM/C, social networks and reference groups are forcing continuation of Type III, and the practice of community social ostracism blocks the abandonment by midwives who refuse to do FGM/C.

Our study confirms the UNFPA [15] findings that the Village Midwives who have taken the oath to cease FGM/C often have to overcome some sanctions while providing anti-FGM/C messages or counselling, such as cultural, economic and religious ones imposed by the society. Midwives often feel constrained and in dilemma by their economic needs, perceptions of FGM/C are cultural importance, and their knowledge of the harm practice poses to girls and women’s health. Therefore, although the midwifery oath aimed to change midwives’ social norms, a conflict may arise between their professional norms and their personal social norms, beliefs, and their sense of “duty” or loyalty to their clients, who request for, and value, FGM/C.

Overall, we have found that changes in FGM/C practice in Sudan include drivers which will not facilitate abandonment of the practice but instead are likely to lead to normalization of FGM/C. The study shows the stark dilemma that despite the clear drivers to reduce Type III FGM/C in Sudan there is a problematic issue with the emphasis on reduction to Type I and not to abandonment. When midwives and families report they believe that Type I ‘is not FGM/C’ it can be used to justify the practice of FGM/C Type 1 continuing. Even when people claimed to have stopped practicing FGM/C, the study suggests they were likely to continue FGM/C Type I.

Worldwide more than 18% of FGM/C procedures are now conducted by health caders [16]. The rates of medicalization, when cutting is performed by a health caders, whether in a home, health facility or elsewhere, vary across countries. Among women aged 15–49 years, the majority (91%) of those who have undergone medicalized FGM/C live in just three countries Egypt, Sudan and Nigeria [10].

In Sudan, medicalization of FGM/C is high as most FGM/C procedures are carried by medical caders, such as midwives, nurses or doctors [7]. A similar pattern was found in our study and the few cases where the services of traditional birth attendants or circumcisers were used may have occurred as a result of the refusal of health care provider, especially midwives, to perform any type of FGM/C.

Globally, most FGM/C is not medicalized, it is still conducted by traditional practitioners (i.e., traditional birth attendants or older women). They fear that failing to have FGM/C will expose their daughters and granddaughters to social stigmatization and reduce their marriageability [4]. FGM/C is also practiced as a rite of passage from childhood to adulthood, a religious obligation, cultural notions such as female cleanliness and beauty [5, 6].

Our study shown that increase in the Medicalization of FGM/C is being driven by families’ belief it may be safer and more hygienic; and the health campaigns that promote the dangers of FGM/C. This confirms recent research [10], indicating that the focus of anti-FGM/C campaigns on the negative health risks of FGM/C may have played a major role in increased medicalization, particularly in places where medicalization is more prevalent. As a less severe form of cutting, Type 1 FGM/C is believed not to cause the health risks associated with the other severe forms of the practice.

Our study has now contributed detailed evidence of further drivers to medicalization which set a research agenda for enquiry in other settings. The strong views that surely a doctor conducting FGM/C would not want to cause harm is likely to be found elsewhere. The preference for medicalized FGM/C by families pressurized socially into having FGM/C also begs further enquiry. The driver of medicalisation in health care providers merely justifying their involvement in FGM/C as response to demand echoes experience in other places along with the widespread availability of health care professionals prepared to do FGM/C. Nonetheless the clear evidence that 3-year-trained Midwives who have taken the professional oath to cease FGM/C are sticking to their word and influencing communities too shows the opportunity to facilitate abandonment rapidly if developed with other Sudan health professionals.

A study by UNFPA [15] on effective deployment and performance of village midwives—the Sudanese health care workers who undertake a one-year certified program and who provide hands-on care at community and health unit level—indicated that the reduction in FGM/C prevalence could be attributed to a newly introduced oath for not practicing FGM/C for these midwives, and the expansion in their training and their wide distribution.

Shift in age of FGM/C was reported by fewer participants in our study. Our results showed variant direction of the shift in age at cutting, where. Some communities reported younger ages of cutting to ‘treat illness’, while some communities favored cutting girls at older age so as to reduce health risks and to prepare girls for marriage, contradicting other studies that have reported a shift towards performing FGM/C at young ages [17]. Further research to understand FGM/C age changes and its implications for interventions may be warranted.

### Limitations

Our study has several limitations, related to the sensitivity of FGM/C issue and the fact that in Sudan, many people still do not feel comfortable taking abut it openly. The fact that one of the states has a law banning FGM/C, some inconsistencies were observed between women’s and health care providers’ reports about medicalized FGM/C. As this study was carried out on two localities in each state, within a limited group of people, with specific contextual drivers, its results are not generalizable to all populations in the two states, and other communities may have similar or different contextual drivers, which remain to be explored in future studies.

## Conclusions

Sudan has a high prevalent of FGM/C and increased levels of medicalization, which is one of the main changes evident in the latest surveys, with the observed changes in the type of FGM/C. Our study findings showed that the shift to less severe cutting in some communities is driven, in part, by social and religious norms, coupled with the increased awareness about the health consequences of FGM/C. Interviews with mothers also suggest that some of them are protective of health care providers who perform FGM/C, ostracising those who do not and pressurising families to continue with FGM/C. These drivers have implications for enforcement of laws targeting providers and communities. Understanding the drivers of the changes has important implications for interventions aimed at promoting the abandonment of FGM/C. The recent Midwives’ oath and subsequent refusal to perform FGM/C, particularly Type III, is also driving the shift to less severe forms of FGM/C and urgently needs to be developed in a similar form with other cadres of health professionals in Sudan.

## Supplementary information

**Additional file 1: Table S1.** Socio-demographic Characteristics of the Study Participants.

## Data Availability

The datasets analyzed during the current study are not yet publicly available but are available from the corresponding author on reasonable request.

## Abbreviations

FGM/C: Female Genital Mutilation/Cutting
MICS: Multiple Indicator Cluster Survey
