# Supplementary material for: Shifts in FGM/C practice in Sudan: communities’ perspectives and drivers
Source: BMC Womens Health. 2019 Dec 30;19:168. doi: 10.1186/s12905-019-0863-6 (PMC6937645; doi:10.1186/s12905-019-0863-6)
Supplement: Supplementary file 1 — Additional file 1: Table S1. Socio-demographic Characteristics of the Study Participants. [file 12905_2019_863_MOESM1_ESM.docx]

**Shifts in FGM/C Practice in Sudan: Communities’ Perspectives and Drivers**

Nafisa Bedri^*1^, Huda Sherfi^1^, Ghada Rudwan^1^, Sara Elhadi^1^, Caroline Kabiru**, and Wafaa Amin^1^

| Participant | Age | | | Educational Level | | | Occupation | | |
| --- | --- | --- | --- | --- | --- | --- | --- | --- | --- |
|  | **Category** | **Frequency** | **%** | **Category** | **Frequency** | **%** | **Category** | **Frequency** | **%** |
| Mothers | 26-40 | 21 | 64 | Illiterate | 13 | 39% | Housewife | 31 | 94% |
|  | 41 & above | 12 | 36 | Khalwa | 0 | 0 | Informal Employee | 1 | 3% |
|  |  |  |  | Primary | 15 | 45% | Formal Employee | 1 | 3% |
|  |  |  |  | Intermediate | 3 | 9% |  |  |  |
|  |  |  |  | Secondary | 2 | 7% |  |  |  |
|  |  |  |  | University & above | 0 | 0 |  |  |  |
|  | Total | 33 | 100% |  | 33 | 100% |  | 33 | 100% |
| Grandmothers | 26-40 | 0 | 0 | Illiterate | 20 | 69% | Housewife | 15 | 52% |
|  | 41 & above | 29 | 100 | Khalwa | 7 | 24% | Informal Employee | 13 | 45% |
|  |  |  |  | Primary | 2 | 7% | Formal Employee | 1 | 3% |
|  |  |  |  | Intermediate | 0 | 0 |  |  |  |
|  |  |  |  | Secondary | 0 | 0 |  |  |  |
|  |  |  |  | University & above | 0 | 0 |  |  |  |
|  | Total | 29 | 100% |  | 29 | 100% |  | 29 | 100% |
| Girls | 11-25 | 30 | 100% | Illiterate | 0 | 0 | Housewife | 10 | 33% |
|  | 26 -40 | 0 | 0 | Khalwa | 0 | 0 | Informal Employee | 0 | 0 |
|  | 41& above | 0 | 0 | Primary | 9 | 30% | Formal Employee | 0 | 0 |
|  |  |  |  | Intermediate | 10 | 33% | Student | 20 | 67% |
|  |  |  |  | Secondary | 11 | 37% |  |  |  |
|  |  |  |  | University & above | 0 | 0 |  |  |  |
|  | Total | 30 | 100% |  | 30 | 100% |  | 30 | 100% |
| Fathers | 26 -40 | 9 | 31% | Illiterate | 5 | 17% | Informal Employee | 24 | 83% |
|  | 41 & above | 20 | 69% | Primary | 10 | 35% | Formal Employee | 5 | 17% |
|  |  |  |  | Intermediate | 5 | 17% |  |  |  |
|  |  |  |  | Secondary | 6 | 21% |  |  |  |
|  |  |  |  | University & above | 3 | 10% |  |  |  |
